# High incidence of multidrug-resistant tuberculosis in Bhutan: A cohort study based on national TB surveillance data

**DOI:** 10.1016/j.ijregi.2024.100471

**Published:** 2024-10-10

**Authors:** Thinley Dorji, Karchung Tshering, Lila Adhikari, Thinley Jamtsho, Pavitra Bhujel, Pema Lhaden, Norelle L. Sherry, Chantel Lin, Kristy Horan, Sonam Wangchuk, Patiyan Andersson, Benjamin P. Howden

**Affiliations:** 1 Kanglung Hospital, Trashigang, Bhutan; 2 Department of Microbiology and Immunology at The Peter Doherty Institute for Infection and Immunity, University of Melbourne, Melbourne, Victoria, Australia; 3 National Tuberculosis Reference Laboratory, Royal Centre for Disease Control (Ministry of Health), Serbithang, Thimphu, Bhutan; 4 Royal Centre for Disease Control (Ministry of Health), Serbithang, Thimphu, Bhutan; 5 Microbiological Diagnostic Unit Public Health Laboratory, Department of Microbiology and Immunology at The Peter Doherty Institute for Infection and Immunity, University of Melbourne, Melbourne, Victoria, Australia; 6 Department of Infectious Diseases & Immunology, Austin Health, Heidelberg, Victoria, Australia; 7 Centre for Pathogen Genomics, University of Melbourne, Melbourne, Victoria, Australia

**Keywords:** Mycobacterium tuberculosis, TB, MDR-TB, Bhutan, High TB burden, Risk factors

## Abstract

**Objectives:**

The emergence of multidrug-resistant tuberculosis (MDR-TB) has made the elimination of TB difficult. Currently, there are limited data on MDR-TB and other drug-resistant TB in Bhutan. We aimed to estimate the incidence and explore the potential risk factors associated with MDR/pre-extensively drug-resistant (pre-XDR)-TB using comprehensive national TB data.

**Methods:**

We used the data from the Tuberculosis Information Surveillance System of the National Tuberculosis Reference Laboratory to analyze the resistance pattern and epidemiologic data for all TB cases tested for resistance for the year 2018-2021. Multiple logistic regression was performed to identify risk factors associated with MDR/pre-XDR-TB.

**Results:**

Of the 2,290 samples tested for drug resistance, 10.6% (n = 243) was MDR-TB, 3.5% (n = 81) was isoniazid-resistant TB, and 0.61% (n = 14) was pre-XDR-TB. A high incidence of MDR/RR-TB/pre-XDR-TB was documented among patients in Thimphu, Samtse, and Sarpang districts. MDR/pre-XDR-TB was more likely documented among patients aged 18-39 years (adjusted odds ratio [aOR] 2.79; 95% confidence interval [CI] 1.46-6.07), female sex (aOR 1.37; 95% CI 1.01-1.86), and patients previously treated for TB (aOR 2.98; 95% CI 1.99-4.42).

**Conclusions:**

Given the high burden of MDR-TB in some districts, decentralization of diagnostic facilities for more rapid characterization could improve early recognition of drug-resistant cases and assist in management. Comprehensive follow-up and monitoring of high-risk groups should be performed.

## Highlights

• High incidence of multidrug-resistant tuberculosis (MDR-TB) in Bhutan was documented.
• Young age, female sex, and previously TB-treatment are at high risk of MDR/pre-extensively drug-resistant-TB.
• MDR/rifampicin-resistant-TB/pre-extensively drug-resistant-TB burden is high in the capital and bordering districts of India.
• Decentralization of testing centers to high-risk areas could improve monitoring.

## Introduction

Globally, the COVID-19 pandemic led to a surge in tuberculosis (TB) cases, reversing years of progress in reducing the burden of disease [1]. Unlike most bacteria, *Mycobacterium tuberculosis* (MTB) is intrinsically resistant to most antibiotics [2], leaving a limited number of drugs available for treatment. Multidrug-resistant TB (MDR-TB) are resistant to at least rifampicin and isoniazid [3]. One of the risk factors for its development is prior exposure to TB drugs [4]. The treatment of MDR-TB involves the use of toxic second-line drugs and is usually associated with lower success rates (63% for MDR-TB vs 88% for drug-sensitive TB [DS-TB]) [3] and higher mortality [5] compared with DS-TB.

An estimated 410,000 cases of MDR/rifampicin-resistant (RR) TB were diagnosed globally in 2022 [3]. Currently, universal drug testing is recommended for all TB cases before treatment [1] to ensure appropriate treatment. The World Health Organization (WHO) has endorsed several rapid diagnostic tests: Xpert MTB/RIF (Cepheid, USA), GenoType MTBDRplus (Hain Lifescience, Germany), and MTBDRsl (Hain Lifescience, Germany) to detect drug resistance. Although Xpert MTB/RIF can detect mutations in the rifampicin resistance determining region (RRDR) of the *rpoB* gene, GenoType MTBDRplus identifies resistance to both rifampicin and isoniazid through the detection of mutations in the *inhA* and *katG* genes with high sensitivity and specificity for cultured isolates [6]. GenoType MTBDRsl detects resistance to fluoroquinolones (levofloxacin, ofloxacin, or moxifloxacin) and second-line injectables (amikacin, kanamycin, or capreomycin) by looking for resistance-conferring mutations in *gyrA* gene and *rrs* gene [6].

As in most developing countries, TB is a leading public health problem in Bhutan, with an incidence rate of 164/100,000 population [7]. Over the past decade, the number of TB notifications has decreased, whereas MDR-TB has increased [8]. An annual drug resistance study in 2014 showed the proportion of primary MDR-TB (MDR-TB among new cases) at 10% and secondary MDR-TB (MDR-TB among previously treated cases) at 37.21% [9]. In 2021, the WHO estimated the proportion of primary MDR/RR-TB at 13% and secondary MDR/RR-TB at 18% [3]. During the same time, the incidence of MDR/RR-TB was estimated to be 170 [3].

Currently, there are few studies on drug-resistant TB (DR-TB) in Bhutan [10,11] and those available focus on a subset of it. Understanding the true burden of DR-TB will help in policy decision-making, planning, and implementation of appropriate interventions, as well as the establishment of additional diagnostic centers in appropriate locations. Therefore, this study aimed to define the resistance pattern of TB in Bhutan and explore the risk factors associated with MDR/pre-extensively drug-resistant (pre-XDR)-TB cases. This would facilitate the formulation of informed evidence-based future interventions.

## Methods

### Study design and settings

This was a retrospective cohort study using the data from the Tuberculosis Information Surveillance System (TBISS) of the National Tuberculosis Reference Laboratory (NTRL), Royal Centre for Disease Control (RCDC), Ministry of Health in Bhutan for the year 2018-2021. The data were collected as part of the routine national TB surveillance system and were de-identified by the data custodians prior to being provided to the analysis team. Population data for respective districts were downloaded from the National Statistics Bureau website (https://www.nsb.gov.bt/publications/annual-dzongkhag-statistics/). Additional population data for rate calculations were sourced from the World Bank dataset (https://data.worldbank.org/indicator/SP.POP.TOTL?locations=BT).

### Sample collection and testing

All patients with presumptive TB provide three sputum (spot, morning, and spot) samples for diagnosis, which are examined using microscopy (46 centers) and Xpert MTB/RIF (currently available at 11 sites, including regional referral hospitals) [8]. Extrapulmonary TB (EPTB) is diagnosed based on clinicopathologic examination and Xpert MTB/RIF.

Smear-positive and presumptive TB samples are shipped to the NTRL, RCDC for genotypic (using MTBDRplus) and phenotypic drug susceptibility tests (pDST). Culture and identification were done as previously described [9]. Subsequently, the culture undergoes pDST using critical concentrations of rifampicin (1 µg/ml), isoniazid (0.1 µg/ml), ethambutol (5 µg/ml), and streptomycin (1 µg/ml) using MGIT or MTBDRplus. Genotypic resistance to fluoroquinolones and second-line injectables is tested using the WHO-recommended GenoType MTBDRsl for MDR-TB.

### Case definition

DR-TB in this study was defined as any drug resistance detected by one of the following tests: Xpert MTB/RIF, MTBDRplus, or pDST. Although pDST was regarded as the gold standard for the diagnosis of DR-TB, genotypic tests were used in its absence. If there was a discrepancy between Xpert MTB/RIF and MTBDRplus, it was classified as resistant.

The resistance pattern was defined as per the updated WHO definition of drug resistance. MDR-TB was a case resistant to at least rifampicin and isoniazid, whereas pre-XDR TB was a case of RR-TB or MDR-TB resistant to fluoroquinolones [3]. Currently, NTRL does not test for bedaquiline or linezolid resistance. In cases where isolates could not be cultured (no growth, contamination, or sample loss), patients with samples testing resistant to rifampicin by Xpert are classified as MDR/RR-TB, consistent with use in the WHO reporting [3].

### Statistical analysis

Data normality for continuous variables were tested using the Shapiro-Wilk test. Categorical data were presented as frequency and proportions. The Kruskal-Wallis test was used to examine differences in age by resistance profile. Trend analysis of MDR-TB by year was performed using the Cochran-Armitage test. All MDR-TB and pre-XDR-TB cases, resistant to at least rifampicin and isoniazid by pDST, MTBDRplus, and MTBDRsl, were grouped as “MDR/pre-XDR-TB” and compared with DS-TB in a backward stepwise multiple logistic regression to identify associated risk factors (age, sex, region, site of TB, type of case, and year of diagnosis). *P*-values less than 0.05 were considered significant. Multicollinearity was tested using the variance inflation factor, and model fitness using the Wald test. All analyses were run using the R-studio (version 2023.12.0), unless stated otherwise.

## Results

**Figure 1:**
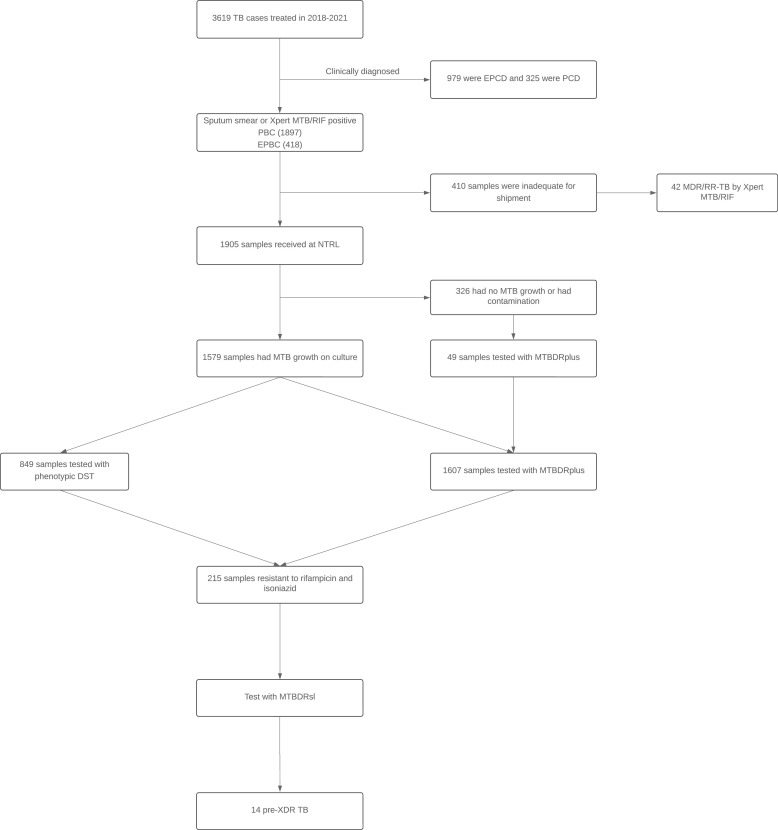
Flowchart of the TB samples tested with different tests during the study period. DST: drug susceptibility testing; EPCD: extra-pulmonary clinically diagnosed; EPBC: extrapulmonary bacteriologically confirmed; MDR-TB: multidrug-resistant TB; MTB: Mycobacterium tuberculosis; NTRL: National Tuberculosis Reference Laboratory; PBC: pulmonary bacteriologically confirmed; PCD: pulmonary clinically diagnosed; TB: tuberculosis.

During the study period, a total of 3,619 cases of TB were diagnosed. Of these, 52.4% (n = 1,897) were pulmonary bacteriologically confirmed TB (PBC), 11.6% (n = 418) were extrapulmonary bacteriologically confirmed TB (EPBC), 9% (n = 325) were pulmonary clinically diagnosed (PCD), and 27.1% (n = 979) were extra-PCD (EPCD). No samples were collected from PCD and EPCD cases, as they were clinically diagnosed based on symptoms. Of the bacteriologically confirmed cases, only 52.6% (n = 1,905) of the samples were received by the NTRL. Culture was positive for 82.9% (n = 1,579) of samples and17.1% (n = 326) had no growth or were contaminated (Figure 1).

### Resistance pattern using pDST

**Figure 2:**
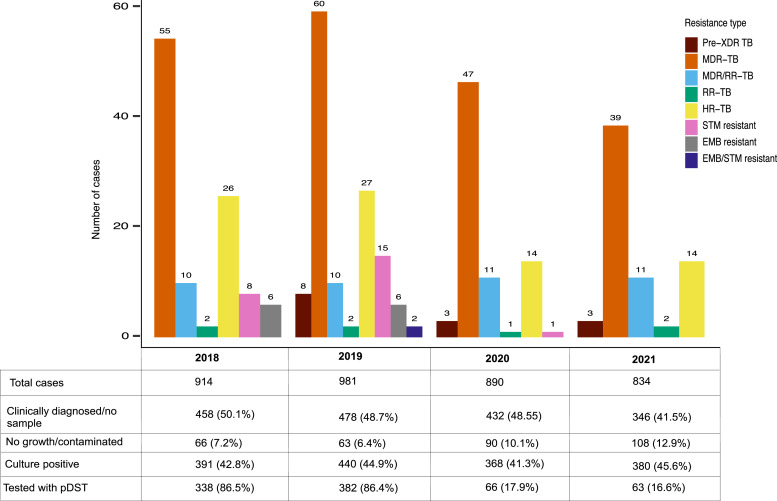
Temporal analysis of different drug resistance profiles from 2018 to 2021. EMB: ethambutol resistant TB; HR-TB: isoniazid-resistant TB; MDR-TB: multidrug-resistant TB; MDR/RR-TB: rifampicin-resistant by Xpert MTB/RIF, isoniazid resistance not confirmed by other test and treated as MDR-TB; pDST: phenotypic drug susceptibility test; pre-XDR TB: pre-extensively drug-resistant TB; RR-TB: rifampicin-resistant TB; STM: streptomycin-resistant TB; TB: tuberculosis. The table below the bar graph shows the number of TB cases for a particular year based on pDST.

Of the cultured samples, only 849 (53.8%) were tested for drug resistance using pDST (Rest tested with MTBDRplus). The proportion of samples tested using pDST differed during the study period, with the highest proportion tested in 2019 (86.5%) with less proportion of samples tested during the pandemic time due to the use of MTBDRplus (Figure 2). Using pDST, 70.2% (n = 596) of samples were sensitive to all first-line drugs. The proportion of MDR-TB was 16% (n = 136), followed by isoniazid-resistant TB (HR-TB) (7.8%, n = 66) and streptomycin-resistant TB (2.8%, n = 24). The proportion of pre-XDR-TB was low (1.1%, n = 9). Excluding eight samples with missing past treatment history, the proportion of primary MDR-TB was 14.4% (107/745) and secondary MDR-TB was 27.1% (26/96).

### Comparison of GeneXpert MTB/RIF with pDST

During the study period, 2,176 (60.1%) samples were tested with Xpert MTB/RIF. Of these, 79.9% (n = 1,738) were inferred as rifampicin susceptible, whereas 11.8% (n = 256) were rifampicin resistant. Three samples were classified as TI (MTB detected, resistance indeterminant), one was indeterminant, and 178 (8.2%) were negative for *MTB*. Xpert MTB/RIF had high sensitivity (97.2%) and high specificity (99.5%) for the detection of rifampicin resistance compared with pDST. Among the Xpert-sensitive samples, five were latter diagnosed to be MDR-TB, 76 were HR-TB, and 35 were resistant to ethambutol or streptomycin using pDST. In addition, nine Xpert-negative samples were culture-positive, including two MDR-TB and one HR-TB.

### Comparison of MTBDRplus with phenotypic drug susceptibility test

A total of 1,607 samples were tested for rifampicin and isoniazid resistance using MTBDRplus. Rifampicin resistance was diagnosed in 13.5% (n = 217) of samples and had a sensitivity of 95.8% and specificity of 99.2% compared with pDST. Isoniazid resistance was detected in 17.2% (n = 276) of samples with a sensitivity of 91.7% and specificity of 99.5%. MDR-TB was identified in 13% (n = 209) of cases, HR-TB in 4.2% (n = 67), and RR-TB in 0.5% (n = 8).

### Incidence of drug-resistant tuberculosis using any method

Of the total TB cases, 63.2% (n = 2,289) were tested for drug resistance by at least one of the above tests. Resistance to any TB drug was observed in 16.7% (383/2,289) (95% confidence interval [CI] 15.2-18.3) of cases. The incidence rate of MDR-TB in this study was 10.6% (243/2,289) (95% CI 9.4-12), including 42 MDR/RR-TB cases. During the same period, the incidence of HR-TB was 3.5% (81/2,289) (95% CI 2.8-4.4) and pre-XDR-TB was 0.61% (14/2,289) (95% CI 0.35-1.05). Among any drug-resistant cases, MDR-TB accounted for 63.4% (n = 243, including MDR/RR-TB), whereas HR-TB accounted for 21.1% (n = 81). Using prior TB treatment history (treatment history not available for 12 patients), the proportion of primary MDR-TB was 9% (182/2,016), and the proportion of secondary MDR-TB was 21.1% (55/261).

### Demographic characteristics of DR-TB

The median age of all patients with any DR-TB was 26 years (range 5-86), with significant differences based on the resistance profiles (Kruskal-Wallis test; *P*-value = 0.032) (Supplementary Table 1). Based on occupation, students/trainees accounted for 25% (n = 50) of all MDR-TB cases, followed by farmers (13%, n = 26) and housewives (10%, n = 21). Moreover, students/trainees also accounted for 43% (n = 6) of all pre-XDR-TB.

### Distribution of drug-resistant TB in Bhutan

**Figure 3:**
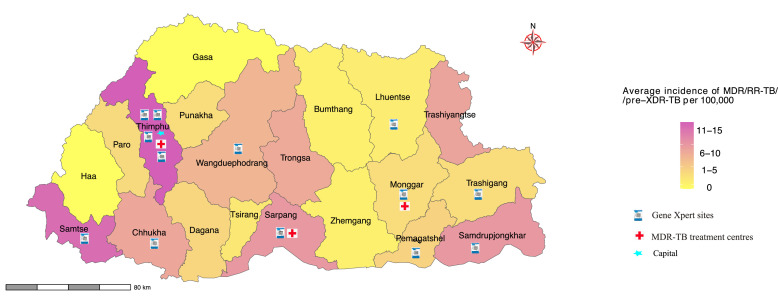
Map of Bhutan showing the average distribution of MDR/RR-TB/pre-XDR TB by districts for the year 2018-2021. Symbols indicate the current MDR-TB treatment centers and Gene Xpert MTB/RIF sites. MDR-TB: multidrug-resistant TB; pre-XDR TB: pre-extensively drug-resistant TB; RR-TB: rifampicin resistant TB; TB: tuberculosis.

Overall, there was a decrease in all types of drug resistance from 2018 to 2021. The incidence of MDR-TB was 12.5% (65/539, including RR/MDR-TB) in 2018, 11.7% (70/598) in 2019, 10% (58/575) in 2020, and 8.7% (50/577) in 2021, with significant decrease over the study period (Cochran-Armitage test; *P*-value = 0.041). There was only one case of streptomycin resistance recorded in 2020 and no cases in 2021, attributed to a decrease in pDST during the COVID pandemic (Figure 2). During the study period, MDR/RR-TB/pre-XDR-TB was predominantly diagnosed among patients living in Thimphu (Capital) and bordering districts of Samtse, Samdrup Jongkhar, and Sarpang districts (Figure 3). The overall incidence of MDR/RR-TB/pre-XDR-TB during the study period was 8.3/100,000 population (8.5/100,000 population in 2018, 10.2/100,000 in 2019, 61/100,000 in 2020, and 6.8/100,000 in 2021).

### Factors associated with MDR/pre-XDR-TB vs DS-TB

Initially, we explored factors associated with MDR-TB and DS-TB using only samples tested by pDST. Backward stepwise multiple logistic regression showed MDR-TB was more likely associated with patients aged <18 years (adjusted odds ratio [aOR]; 3.53, 95% confidence interval [CI] 1.15-11.8, *P*-value 0.031), 18-39 years (aOR 3.93; 95% CI 1.68-11.0; *P*-value 0.004), patients previously treated for TB (aOR 1.93; 95% CI 1.11-3.28; *P*-value 0.017), and TB diagnosed in 2021 (aOR 8.31; 95% CI 4.26-16.6, *P*-value <0.001) (Table 1).

**Table 1:** Factors associated with MDR-TB compared with drug-sensitive TB (by phenotypic drug susceptibility test).

Characteristic	Proportion of cases		Unadjusted			Adjusted		
	DS-TB, N = 596^a^	MDR-TB, N = 145^a^	OR^b^	95% CI^b^	*P*-value	OR^b^	95% CI^b^	*P*-value
**Age**								
<18 years	49 (8.2%)	10 (6.9%)	2.14	0.74, 6.68	0.2	3.53	1.15, 11.8	0.031^c^
18-39 years	399 (67%)	111 (77%)	2.82	1.28, 7.46	0.019^c^	3.93	1.68, 11.0	0.004^c^
40-59 years	85 (14%)	18 (12%)	2.25	0.89, 6.50	0.10	2.62	0.97, 8.05	0.070
≥60 years	63 (11%)	6 (4.1%)	—	—		—	—	
**Sex**								
Female	299 (50%)	85 (59%)	1.38	0.95, 2.01	0.089			
Male	297 (50%)	60 (41%)	—	—				
**Region**								
Western region	481 (81%)	125 (86%)	1.65	0.92, 3.18	0.11			
Central region	84 (14%)	14 (9.7%)	—	—				
Eastern region	31 (5.2%)	6 (4.1%)	1.25	0.41, 3.47	0.7			
**Site of infection**								
PBC	569 (95%)	131 (90%)	—	—				
EPBC	27 (4.5%)	14 (9.7%)	2.31	1.12, 4.57	0.018^c^			
**Type of case**								
New	533 (89%)	114 (79%)	—	—		—	—	
Previously Treated	61 (10%)	27 (19%)	2.07	1.24, 3.37	0.004^c^	1.93	1.11, 3.28	0.017^c^
Missing	2 (0.3%)	4 (2.8%)						
**Year of diagnosis**								
2018	250 (42%)	46 (32%)	—	—		—	—	
2019	276 (46%)	60 (41%)	1.14	0.74, 1.75	0.6	1.13	0.73, 1.74	0.6
2020	49 (8.2%)	10 (6.9%)	1.12	0.51, 2.30	0.8	1.09	0.49, 2.26	0.8
2021	21 (3.5%)	29 (20%)	7.61	4.02, 14.7	<0.001^c^	8.31	4.26, 16.6	<0.001^c^

a n (%)

b OR = odds ratio, CI = confidence interval

c *P*-value <0.05. EPBC, extrapulmonary bacteriologically confirmed; DS-TB, drug-sensitive TB; MDR-TB, multidrug-resistant TB; PBC, pulmonary bacteriologically confirmed; TB, tuberculosis.

Second, we assessed the association between MDR/pre-XDR-TB and DS-TB using samples tested by pDST and MTBDRplus, as all samples were not tested using pDST (Table 2). MDR/pre-XDR-TB was more likely to be diagnosed among patients aged 18-39 years (aOR 2.79; 95% CI 1.46-6.07; *P*-value 0.004), female sex (aOR 1.37; 95% CI 1.01-1.86; *P*-value 0.047), and patients previously treated for TB (aOR 2.98; 95% CI 1.99-4.42; *P*-value <0.001). We also tested for the association between MDR/pre-XDR-TB and other DR-TB (Supplementary Table 2). Female sex (aOR 2.29; 95% CI 1.44-3.68; *P*-value < 0.01), EPBC (aOR 3.79; 05% CI 1.54-11.4; *P*-value 0.008), previously treated for TB (aOR 2.79; 95% CI 1.39-6.04; *P*-value 0.006), diagnosed in 2020 (aOR 2.71; 95% CI 1.34-5.67; *P*-value 0.006), and diagnosed in 2021 (aOR 2.15; 95% CI 1.05-4.58; *P*-value 0.04) were more likely to have MDR/pre-XDR-TB.

**Table 2:** Factors associated with MDR/pre-XDR-TB compared with DS-TB (tested using pDST, MTBDRplus, and MTBDRsl).

Characteristic	Proportion of cases		Unadjusted			Adjusted		
	DS-TB, N = 1286^a^	MDR/pre-XDR-TB, N = 215^a^	OR^b^	95% CI^b^	*P*-value	OR^b^	95% CI^b^	*P*-value
**Age**								
<18 years	99 (7.7%)	11 (5.1%)	1.77	0.71, 4.53	0.2	1.80	0.71, 4.69	0.2
18-39 years	830 (65%)	162 (75%)	3.01	1.59, 6.47	0.002^c^	2.79	1.46, 6.07	0.004^c^
40-59 years	214 (17%)	33 (15%)	2.39	1.15, 5.45	0.027^c^	2.10	1.00, 4.82	0.062
≥60 years	143 (11%)	9 (4.2%)	—	—		—	—	
**Sex**								
Female	637 (50%)	125 (58%)	1.43	1.06, 1.93	0.018^c^	1.37	1.01, 1.86	0.047^c^
Male	649 (50%)	90 (42%)	—	—		—	—	
**Region**								
Western region	1,039 (81%)	185 (86%)	1.48	0.94, 2.45	0.11			
Central region	178 (14%)	23 (11%)	—	—				
Eastern region	69 (5.4%)	7 (3.3%)	0.86	0.33, 2.03	0.7			
**Site of infection**								
PBC	1,208 (94%)	192 (89%)	—	—		—	—	
EPBC	78 (6.1%)	23 (11%)	1.87	1.11, 3.03	0.014^c^	1.57	0.92, 2.58	0.085
**Type of case**								
New	1,175 (91%)	165 (77%)	—	—		—	—	
Previously Treated	109 (8.5%)	44 (20%)	2.87	1.94, 4.21	<0.001^c^	2.98	1.99, 4.42	<0.001^c^
Missing	2 (0.2%)	6 (2.8%)						
**Year of diagnosis**								
2018	295 (23%)	55 (26%)	—	—				
2019	328 (26%)	68 (32%)	1.08	0.73, 1.61	0.7			
2021	338 (26%)	42 (20%)	0.69	0.44, 1.06	0.090			
2020	325 (25%)	50 (23%)	0.85	0.56, 1.29	0.4			

a n (%)

b OR = odds ratio, CI = confidence interval

c *P*-value <0.05. EPBC, extrapulmonary bacteriologically confirmed; DS-TB, drug-sensitive TB; MDR-TB, multidrug-resistant TB; Pre-XDR-TB, pre-extensively drug-resistant TB; PBC, pulmonary bacteriologically confirmed TB; pDST, phenotypic drug susceptibility testing; TB, tuberculosis.

## Discussion

The aim of this study was to estimate the incidence of MDR-TB in Bhutan using comprehensive national TB data and identify the risk factors associated with MDR/pre-XDR-TB compared with DS-TB. Our study showed a high incidence of MDR-TB with a low incidence of HR-TB and pre-XDR-TB. Furthermore, MDR/pre-XDR-TB was more likely to be seen among young adults, females, and patients previously treated for TB compared with DS-TB.

During the COVID-19 pandemic, there was a global resurgence in TB, including MDR-TB [1]. However, a smaller number of MDR-TB compared with pre-pandemic were diagnosed in Bhutan. One potential reason could be reduced TB transmission due to movement restrictions, public gatherings, social distancing, and the use of face masks. However, another plausible reason could be the disruption of TB services, as evidenced by decreased TB case detection [12] and reflected by the decreased pDST testing during the pandemic. Similar changes have been observed in other high-burden countries [13]. A retrospective study in Nigeria showed decreased notification of TB, including RR-TB, during the pandemic, followed by increased notification of RR-TB post-COVID [14]. Nevertheless, preventive measures such as BCG vaccination remained high even during the pandemic in Bhutan [15].

In this study, 63% of any cases with DR-TB had a MDR-TB pattern, followed by HR-TB pattern. Previous studies in Bhutan also confirmed MDR-TB to be the most common resistance, followed by HR-TB [11]. This contrasts with findings from neighboring countries of India [16,17] and Thailand [18], where HR-TB is the most common DR-TB. The reason for the high proportion of MDR-TB in Bhutan compared with other neighboring countries is not known. There could be different variants of MDR-TB in Bhutan with high transmissibility, or the difference could be due to different demographic factors in Bhutan.

The proportion of primary MDR-TB in Bhutan (9%) is higher than the global percentage of 3.6 % [1] and from neighboring countries of India (3.5%) [17] and Thailand (0.8%) [18]. However, this is lower than the 13% estimated by the WHO [3]. Conversely, the MDR-TB among the previously treated cases (21.1%) was higher than the WHO estimates (18%) in 2021 [3] but lower than in India (26.7%) [17]. This could signify that the major contributor of MDR-TB in Bhutan is due to the active transmission of resistant strains in the community. This is supported by previous studies, which showed low default rates and treatment failures [19], which are risk factors for the development of drug resistance. Further studies to explore the genetic relationship of cases with MDR-TB through whole genome sequencing would provide further insights into the dynamics of MDR-TB evolution and transmission in Bhutan.

Most studies that report MDR-TB have not tested for fluoroquinolone resistance, and therefore, it cannot be excluded that a subset of this may be pre-XDR-TB. Therefore, we have compared our findings of MDR/pre-XDR-TB to MDR-TB in other studies. Consistent with our findings, other studies have indicated that previous exposure to TB drugs is associated with MDR-TB [4]. Drug resistance in previously treated patients can occur due to *de novo* mutations acquired during treatment. However, yet other studies have shown no association with previous TB treatment [16]. Nonetheless, the burden of MDR-TB is driven mainly by the transmission of the resistant strains [20]. This is true even among previously treated patients, where transmission accounts for a major proportion of MDR-TB [21].

Female sex was associated with MDR-TB, consistent with studies from South India and Georgia [4,16]. In contrast, studies from Africa showed no association between sex and MDR-TB [22]. This association could be explained by the social responsibilities of women in Bhutan to provide care for people with MDR-TB, similar to other countries [4].

Younger patients are more likely to be diagnosed with MDR-TB as observed in other studies [16,23]. One of the plausible reasons could be the high mobility of the younger age groups, which results in an increased risk of contact with other patients with MDR-TB [23]. Moreover, people who drink alcohol and smoke cigarettes are at higher risk of MDR-TB [24], and these behaviors are more common among young people in Bhutan compared with others [25].

In agreement with other studies [26], Xpert MTB/RIF had high sensitivity and specificity in detecting rifampicin resistance in Bhutan. This ensures that cases with MDR/RR-TB are diagnosed early and commenced on effective treatment, thereby improving the patient treatment outcomes as well as interruption of the transmission chain. Installation of Xpert MTB/RIF does not entail huge capital investment and can easily be used in field hospitals with minimal training. Furthermore, the results can be delivered with a turn-around time of 2 hours as it is tested on clinical samples. Such diagnostic technologies are even more relevant and helpful for Bhutan as sample shipment to referral centers takes days due to difficult geographical terrain and inefficient transportation systems, which further delays the turn-around time for detection.

This study highlights five MDR-TB missed by Xpert MTB/RIF, leading to ineffective treatment with first-line drugs. This further exacerbates the resistance and transmission. This is one of the limitations of Xpert MTB/RIF as it only interrogates mutations present in rifampicin resistance determining region (codon 426-452) of *rpoB* gene [27]. Rifampicin resistance-conferring mutations are known to occur outside this region, especially in *rpoB* V170F and I491F [27]. Studies in South Africa have demonstrated outbreaks of MDR-TB due to the presence of mutations occurring outside the RRDR and missed by Xpert MTB/RIF [28]. Although the Xpert MTB/RIF has high sensitivity and specificity for the diagnosis of PTB, it has variable sensitivity and specificity for EPTB [29] and low sensitivity (77.7%) for the detection of TB in smear-negative culture-positive samples [26]. This was evident in our study, where nine cases with presumptive smear-negative TB were missed by Xpert.

Thirty-six percent of our cases were clinically diagnosed and a proportion of these could have potentially grown *MTB* on culture. A study from India reported culture positivity of 8% among smear-negative TB and 6% among EPTB [30]. Moreover, it is likely that some presumptive cases would have been misdiagnosed as non-TB on smear microscopy. The development of a comprehensive guideline for culturing smear-negative and Xpert negative presumptive cases by NTRL could alleviate the diagnosis gap.

Overall, our results showed just over half of the cases had their samples cultured, and only 50% of these were tested for resistance using pDST, which is attributed to prolonged transit times for samples shipped from hospitals to NTRL, which invariably compromises cold chain. Presently, TB diagnosis in most peripheral hospitals relies on smear microscopy and chest x-ray, as the logistical challenges pose a significant barrier to the uptake of centralized phenotypic susceptibility testing, and results are not available in a clinically relevant time limit to influence patient management. Further, prolonged storage of samples before shipment has compromised viability and increased contamination. Therefore, the National Tuberculosis Control Program and NTRL should explore decentralization and the introduction of culture and testing centers in regional MDR-TB centers. GenoType MTBDRplus, which can be performed on clinical samples, could be employed in high-burden districts to enhance the detection of MDR-TB, taking advantage of molecular testing capacity established in regional referral hospitals during the COVID-19 pandemic.

Whole genome sequencing for TB has not been performed in Bhutan at this stage, and there is no current knowledge on circulating resistance mutations and lineages in Bhutan. Given the differences in MDR-TB compared with other countries, we believe that it is critical that whole genome sequencing capacity is built at the NTRL, as implementation of whole genome sequencing will ensure timely provision of drug resistance results for all TB drugs and help in understanding the dynamics of TB transmission. Understanding the risk factors for the acquisition of MDR-TB in the Bhutanese setting, including travel history, living/studying overseas, and genomic relationships between strains (to identify the likelihood of local transmission vs introduction from overseas) will be important to inform potential public health interventions. This is especially vital as Bhutan strives to achieve the targets of the End TB strategy by 2035.

### Limitations

The pDST is the current gold standard for the diagnosis of DR-TB. However, samples were not collected for all patients, as many were diagnosed and treated based on clinical signs. Where samples were available, a substantial proportion of samples were contaminated, did not grow a viable MTB, or were lost during the sample shipment from district hospitals to NTRL. Moreover, amidst the COVID pandemic, shipment of samples was hampered and those that reached underwent testing with only MTBDRplus due to faster turn-around times. Consequently, the resistance pattern may have been different if pDST was used due to the interrogation of limited genomic region by MTBDRplus. Furthermore, there are additional risk factors not available in our dataset that could affect the incidence of MDR-TB.

Moreover, our data only includes notified TB cases based on passive screening. It is likely that there would be a greater number of TB cases, including MDR-TB, who are asymptomatic or undiagnosed, as shown by the low TB case detection rate of Bhutan of 67% in 2021 [12].

## Conclusion

This study demonstrates a high incidence of MDR-TB alongside a low incidence of isoniazid-resistant TB in Bhutan. The distribution of MDR-TB is disproportionate, indicating a need for decentralizing diagnostic tests like MTBDRplus to areas with a high burden of MDR-TB. There is an opportunity for NTRL to explore the utility of genomics to understand the transmission dynamics of TB in Bhutan.

## Declarations of competing interest

The authors have no competing interests to declare.

## Appendix B Supplementary materials

Image, application 1
