# Supplementary material for: High incidence of multidrug-resistant tuberculosis in Bhutan: A cohort study based on national TB surveillance data
Source: IJID Reg. 2024 Oct 10;13:100471. doi: 10.1016/j.ijregi.2024.100471 (PMC11541819; doi:10.1016/j.ijregi.2024.100471)
Supplement: Supplementary file 1 [file mmc1.docx]

**Supplementary table**

Supplementary table 1. Demographic and clinical characteristics of all types of drug-resistant cases.

| **Characteristic** | **Overall**, N = 383^1^ | **EMB resistant**, N = 12^1^ | **EMB/STM resistant**, N = 2^1^ | **HR-TB**, N = 81^1^ | **MDR-TB**, N = 201^1^ | **Pre-XDR TB**, N = 14^1^ | **RR-TB**, N = 7^1^ | **RR/MDR-TB**, N = 42^1^ | **STM resistant**, N = 24^1^ |
| --- | --- | --- | --- | --- | --- | --- | --- | --- | --- |
| **Age (Range)** | 26 (22, 35) | 24 (20, 36) | 41 (31, 52) | 26 (22, 34) | 26 (22, 33) | 26 (19, 39) | 33 (29, 49) | 24 (21, 31) | 38 (25, 51) |
| **Age** |  |  |  |  |  |  |  |  |  |
| <18 years | 22 (5·7%) | 2 (17%) | 0 (0%) | 3 (3·7%) | 10 (5·0%) | 1 (7·1%) | 0 (0%) | 5 (12%) | 1 (4·2%) |
| 18-39 years | 281 (73%) | 7 (58%) | 1 (50%) | 62 (77%) | 153 (76%) | 9 (64%) | 5 (71%) | 33 (79%) | 11 (46%) |
| 40-59 years | 58 (15%) | 0 (0%) | 0 (0%) | 11 (14%) | 30 (15%) | 3 (21%) | 0 (0%) | 3 (7·1%) | 11 (46%) |
| ≥ 60 years | 22 (5·7%) | 3 (25%) | 1 (50%) | 5 (6·2%) | 8 (4·0%) | 1 (7·1%) | 2 (29%) | 1 (2·4%) | 1 (4·2%) |
| **Gender** |  |  |  |  |  |  |  |  |  |
| Female | 205 (54%) | 5 (42%) | 0 (0%) | 34 (42%) | 120 (60%) | 5 (36%) | 2 (29%) | 32 (76%) | 7 (29%) |
| Male | 178 (46%) | 7 (58%) | 2 (100%) | 47 (58%) | 81 (40%) | 9 (64%) | 5 (71%) | 10 (24%) | 17 (71%) |
| **Occupation of the patients** |  |  |  |  |  |  |  |  |  |
|  |  |  |  |  |  |  |  |  |  |
| Student/Trainee | 92 (24%) | 3 (25%) | 1 (50%) | 20 (25%) | 50 (25%) | 6 (43%) | 1 (14%) | 9 (21%) | 2 (8·3%) |
| Farmer | 56 (15%) | 2 (17%) | 1 (50%) | 14 (17%) | 26 (13%) | 0 (0%) | 2 (29%) | 3 (7·1%) | 8 (33%) |
| Private /Business | 39 (10%) | 1 (8·3%) | 0 (0%) | 10 (12%) | 16 (8·0%) | 5 (36%) | 1 (14%) | 4 (9·5%) | 2 (8·3%) |
| Housewife | 38 (9·9%) | 1 (8·3%) | 0 (0%) | 7 (8·6%) | 21 (10%) | 0 (0%) | 0 (0%) | 6 (14%) | 3 (13%) |
| Civil Servant | 19 (5·0%) | 1 (8·3%) | 0 (0%) | 5 (6·2%) | 12 (6·0%) | 0 (0%) | 0 (0%) | 1 (2·4%) | 0 (0%) |
| Driver | 11 (2·9%) | 0 (0%) | 0 (0%) | 1 (1·2%) | 7 (3·5%) | 0 (0%) | 1 (14%) | 1 (2·4%) | 1 (4·2%) |
| Corporate employee | 6 (1·6%) | 1 (8·3%) | 0 (0%) | 0 (0%) | 4 (2·0%) | 0 (0%) | 0 (0%) | 0 (0%) | 1 (4·2%) |
| Monk/Nun | 6 (1·6%) | 0 (0%) | 0 (0%) | 1 (1·2%) | 4 (2·0%) | 1 (7·1%) | 0 (0%) | 0 (0%) | 0 (0%) |
| Armed forces | 5 (1·3%) | 0 (0%) | 0 (0%) | 0 (0%) | 4 (2·0%) | 0 (0%) | 0 (0%) | 0 (0%) | 1 (4·2%) |
| Dependent | 5 (1·3%) | 1 (8·3%) | 0 (0%) | 1 (1·2%) | 3 (1·5%) | 0 (0%) | 0 (0%) | 0 (0%) | 0 (0%) |
| Retiree | 3 (0·8%) | 1 (8·3%) | 0 (0%) | 2 (2·5%) | 0 (0%) | 0 (0%) | 0 (0%) | 0 (0%) | 0 (0%) |
| Minors | 1 (0·3%) | 0 (0%) | 0 (0%) | 0 (0%) | 1 (0·5%) | 0 (0%) | 0 (0%) | 0 (0%) | 0 (0%) |
| Others | 102 (27%) | 1 (8·3%) | 0 (0%) | 20 (25%) | 53 (26%) | 2 (14%) | 2 (29%) | 18 (43%) | 6 (25%) |
| **Region** |  |  |  |  |  |  |  |  |  |
| Western region | 323 (84%) | 11 (92%) | 2 (100%) | 62 (77%) | 173 (86%) | 12 (86%) | 7 (100%) | 38 (90%) | 18 (75%) |
| Central region | 43 (11%) | 1 (8·3%) | 0 (0%) | 12 (15%) | 21 (10%) | 2 (14%) | 0 (0%) | 3 (7·1%) | 4 (17%) |
| Eastern region | 17 (4·4%) | 0 (0%) | 0 (0%) | 7 (8·6%) | 7 (3·5%) | 0 (0%) | 0 (0%) | 1 (2·4%) | 2 (8·3%) |
| **Site of infection** |  |  |  |  |  |  |  |  |  |
| PBC | 328 (86%) | 12 (100%) | 2 (100%) | 76 (94%) | 179 (89%) | 13 (93%) | 7 (100%) | 15 (36%) | 24 (100%) |
| EPBC | 55 (14%) | 0 (0%) | 0 (0%) | 5 (6·2%) | 22 (11%) | 1 (7·1%) | 0 (0%) | 27 (64%) | 0 (0%) |
| **Treatment history** |  |  |  |  |  |  |  |  |  |
| New | 304 (79%) | 10 (83%) | 2 (100%) | 73 (90%) | 155 (77%) | 10 (71%) | 5 (71%) | 27 (64%) | 22 (92%) |
| Previously Treated | 68 (18%) | 2 (17%) | 0 (0%) | 6 (7·4%) | 42 (21%) | 2 (14%) | 1 (14%) | 13 (31%) | 2 (8·3%) |
| Missing | 11 (2·9%) | 0 (0%) | 0 (0%) | 2 (2·5%) | 4 (2·0%) | 2 (14%) | 1 (14%) | 2 (4·8%) | 0 (0%) |
| **Year of diagnosis** |  |  |  |  |  |  |  |  |  |
| 2018 | 107 (28%) | 6 (50%) | 0 (0%) | 26 (32%) | 55 (27%) | 0 (0%) | 2 (29%) | 10 (24%) | 8 (33%) |
| 2019 | 130 (34%) | 6 (50%) | 2 (100%) | 27 (33%) | 60 (30%) | 8 (57%) | 2 (29%) | 10 (24%) | 15 (63%) |
| 2020 | 77 (20%) | 0 (0%) | 0 (0%) | 14 (17%) | 47 (23%) | 3 (21%) | 1 (14%) | 11 (26%) | 1 (4·2%) |
| 2021 | 69 (18%) | 0 (0%) | 0 (0%) | 14 (17%) | 39 (19%) | 3 (21%) | 2 (29%) | 11 (26%) | 0 (0%) |
| ^1^Median for Age; n (%) | | | | | | | | | |

PBC (pulmonary bacteriologically confirmed TB); EPBC (extra-pulmonary bacteriologically confirmed TB), EMB resistant (ethambutol resistant TB), HR-TB (Isoniazid resistant TB), MDR-TB (Multidrug-resistant TB), pre-XDR-TB (pre-extensively drug-resistant TB), RR-TB (rifampicin resistant TB), STM resistant (streptomycin resistant TB)

Supplementary table 2. Factors associated with MDR/pre-XDR-TB compared to non-MDR-TB cases

|  | **Proportion of cases** | | **Unadjusted** | | | **Adjusted** | | |
| --- | --- | --- | --- | --- | --- | --- | --- | --- |
| **Characteristic** | **MDR/Pre-XDR-TB**, N = 257^1^ | **Non-MDR-TB**, N = 126^1^ | **OR**^2^ | **95% CI**^2^ | **p-value** | **OR**^2^ | **95% CI**^2^ | **p-value** |
| **Age** |  |  |  |  |  |  |  |  |
| <18 years | 16 (6·2%) | 6 (4·8%) | — | — |  |  |  |  |
| 18-39 years | 195 (76%) | 86 (68%) | 0·68 | 0·22, 1·81 | 0·5 |  |  |  |
| 40-59 years | 36 (14%) | 22 (17%) | 0·52 | 0·15, 1·55 | 0·3 |  |  |  |
| ≥ 60 years | 10 (3·9%) | 12 (9·5%) | 0·28 | 0·07, 1·02 | 0·062 |  |  |  |
| **Gender** |  |  |  |  |  |  |  |  |
| Female | 157 (61%) | 48 (38%) | 2·53 | 1·63, 3·97 | <0·001* | 2·29 | 1·44, 3·68 | <0·001* |
| Male | 100 (39%) | 78 (62%) | — | — |  | — | — |  |
| **Region** |  |  |  |  |  |  |  |  |
| Central region | 26 (10%) | 17 (13%) | — | — |  |  |  |  |
| Eastern region | 8 (3·1%) | 9 (7·1%) | 0·71 | 0·22, 2·28 | 0·6 |  |  |  |
| Western region | 223 (87%) | 100 (79%) | 1·57 | 0·80, 3·04 | 0·2 |  |  |  |
| **Site of infection** |  |  |  |  |  |  |  |  |
| EPBC | 50 (19%) | 5 (4·0%) | 5·49 | 2·33, 16·2 | <0·001* | 3·79 | 1·54, 11·4 | 0·008* |
| PBC | 207 (81%) | 121 (96%) | — | — |  | — | — |  |
| **Type of case** |  |  |  |  |  |  |  |  |
| Missing | 8 (3·1%) | 3 (2·4%) |  |  |  |  |  |  |
| New | 192 (75%) | 112 (89%) | — | — |  | — | — |  |
| Previously Treated | 57 (22%) | 11 (8·7%) | 3·02 | 1·58, 6·30 | 0·002* | 2·79 | 1·39, 6·04 | 0·006* |
| **Year of diagnosis** |  |  |  |  |  |  |  |  |
| 2018 | 65 (25%) | 42 (33%) | — | — |  | — | — |  |
| 2019 | 78 (30%) | 52 (41%) | 0·95 | 0·56, 1·62 | 0·9 | 1·10 | 0·63, 1·93 | 0·7 |
| 2020 | 61 (24%) | 16 (13%) | 2·67 | 1·36, 5·43 | 0·005* | 2·71 | 1·34, 5·67 | 0·006* |
| 2021 | 53 (21%) | 16 (13%) | 2·36 | 1·20, 4·82 | 0·015* | 2·15 | 1·05, 4·58 | 0·040* |

^1^n (%) *p-value < 0·05, ^2^OR = Odds Ratio, CI = Confidence Interval, PBC (Pulmonary bacteriologically confirmed TB), EPBC (Extra-pulmonary bacteriologically confirmed TB), pDST (Phenotypic drug susceptibility testing), DS-TB (Drug sensitive TB), MDR-TB (Multidrug-resistant TB), pre-XDR-TB (pre-extensively drug-resistant TB).

Non-MDR cases refers to drug-resistant TB cases other than MDR-TB and pre-XDR-TB.
